# Volumetric evaluation of the sphenoid sinus among different races in the Southeast Asian (SEA) population: a computerized tomography study

**DOI:** 10.7150/ijms.68095

**Published:** 2023-01-16

**Authors:** Geng Ju Tuang, Farah Dayana Zahedi, Salina Husain, Aneeza Khairiyah Wan Hamizan, Thean Yean Kew, Jegan Thanabalan

**Affiliations:** 1 Department of Otorhinolaryngology - Head & Neck Surgery, Faculty of Medicine, Universiti Kebangsaan Malaysia, Kuala Lumpur, Malaysia.; 2 Department of Otorhinolaryngology - Head & Neck Surgery, Hospital Seberang Jaya, Penang, Malaysia.; 3 Department of Radiology, Faculty of Medicine, Universiti Kebangsaan Malaysia, Kuala Lumpur, Malaysia.; 4 Department of Surgery, Faculty of Medicine, Universiti Kebangsaan Malaysia, Kuala Lumpur, Malaysia.

**Keywords:** sphenoid sinus, volume, computed tomography, x-ray, ethnic groups, sex

## Abstract

**Introduction:** The fundament of forensic science lies in identifying a body. The morphological complexity of the paranasal sinus (PNS), which varies greatly amongst individual, possess a discriminatory value that potentially contributes to the radiological identification. The sphenoid bone represents the keystone of the skull and forms part of the cranial vault. It is intimately associated with vital neurovascular structures. The sphenoid sinus, located within the body of the sphenoid bone, has variable morphology. The sphenoid septum's inconsistent position and the degree, as well as the direction disparities of sinus pneumatization, have indeed accorded it a unique structure in providing invaluable information in forensic personnel identification. Additionally, the sphenoid sinus is situated deep within the sphenoid bone. Therefore, it is well protected from traumatic degradation from external causes and can be potentially utilized in forensic studies. The authors aim to study the possibility of variation among the race, and gender in the Southeast Asian (SEA) population, using volumetric measurements of the sphenoid sinus.

**Materials and methods:** This is a retrospective cross-sectional analysis of computerized tomographic (CT) imaging of the PNS of 304 patients (167 males, 137 females) in a single centre. The volume of the sphenoid sinus was reconstructed and measured using commercial real-time segmentation software.

**Result:** The total volume of sphenoid sinus of male gender had shown to be larger, 12.22 (4.93 - 21.09) cm^3^ compared to the counterpart of 10.19 (3.75 - 18.72) cm^3^ (*p* = .0090). The Chinese possessed a larger total sphenoid sinus volume, 12.96 (4.62 - 22.21) cm^3^) than the Malays, 10.68 (4.13 - 19.25) cm^3^ (*p* = .0057). No correlation was identified between the age and volume of the sinus (cc= -.026, *p* = .6559).

**Conclusion:** The sphenoid sinus volume in males was found to be larger than those of females. It was also shown that race influences sinus volume. Volumetric analysis of the sphenoid sinus can potentially be utilized in gender and race determination. The current study provided normative data on the sphenoid sinus volume in the SEA region, which can be helpful for future studies.

## Introduction

The sphenoid bone represents the keystone of the skull and forms part of the cranial vault. It is devoid of air at birth, with a gradual formation of the sphenoid sinus postnatally through the invagination of the nasal mucosa into the posterior portion of paired cartilaginous nasal capsule. Pneumatization of the sphenoid sinus then progresses with age in an inferior posterolateral direction with variant degrees and is intimately associated with vital neurovascular structures 1.

The fundament of forensic science lies within the identification of a body. In a well-preserved body or during the early stage of decomposition, Deoxyribonucleic acid (DNA), profiling and fingerprinting have been the primary method to identify the diseased. On the other hand, the study of anthropometric characteristics and anatomical peculiarities plays an indispensable role in more advanced putrefaction stages or when the DNA is severely degraded 2. The utilization of PNS to identify unknown descendent is well-established in anthropology studies. The morphological complexity of the PNS, which varies greatly amongst individual, possess a discriminatory value that contributes to the radiological identification 3,4.

Much attention has evolved around the frontal and maxillary sinus 2,3,4. The analysis of both sinuses yielded credible evidence in the literature as a secure method for comparative radiography studies that promote human identification 2,3,4,5,6,7. Besides a high sensitivity (83.75%) and specificity (100%), Ruder et al. found a high negative predictive value (95.4%) and positive predictive value (100%) in identifying the matching pairs by comparing the morphology of the PNS of 100 post-mortem to 25 ante-mortem head CT 4. The maxillary sinus volume is found to be helpful in gender determination. Bangi et al. retrospectively measured the dimensions and volume of the maxillary sinus of 100 individuals and observed an accuracy rate of 88% in gender determination based on the two parameters 8. Xavier et al. reviewed 30 articles regarding the application of frontal and maxillary sinus for human identification and sex determination. He concluded a potential utilization of both the frontal and maxillary sinus in human identification, owing to the unique morphological variation of PNS among individuals. The volume and dimension of the maxilla sinus were shown to be significantly higher in males than in females 3. In the context of violent trauma, however, the anatomical position of the frontal and maxillary sinus is vulnerable to severe damage. Shattered pieces of the sinuses or missing teeth may limit their contribution to identification 6.

The sphenoid sinus is intimately related to the surrounding vital neurovascular structures such as the optic nerves, pterygoid nerve, pituitary gland, and carotid arteries 6. The inconsistent position of the sphenoid septum, along with the degree and direction disparities of sinus pneumatization, has indeed accorded the sphenoid sinus a unique structure in providing invaluable information in forensic personnel identification. The degree of pneumatization of the sphenoid sinus ranges from absence or poor (conchal type) to hyperextension beyond the basal surface of the sphenoid bone, with potential involvement of the anterior and posterior clinoid processes, the lesser and greater sphenoid wings, the pterygoid process and plates, and into the clivus 9. Additionally, the deep anatomical location of the sphenoid sinus within the sphenoid body is well protected from traumatic degradation resulting from an external cause 6,10.

The analysis with regards to the sphenoid sinus in many studies described variation and highlighted their findings in their respective populations. Most of these results, however, are based on the Western population 6,10-14. The authors aim to study the possibility of variation among the race, and gender in the SEA population, utilizing a volumetric measurement of the sphenoid sinus.

## Materials and methods

This study consists of a cross-sectional retrospective evaluation of multiplanar images (three planes: axial, coronal, and sagittal) of all patients who underwent CT PNS at a tertiary referral centre in Malaysia over three years (2016 - 2019). Approval for this study was obtained from the ethical committee of the institute. (UKM PPI/111/8/JEP-2019-734).

Patients with a history of skull base surgery, previous functional endoscopic sinus surgery, extensive nasal polyposis, traumatic facial bone and PNS fracture, and paranasal neoplasm were excluded from the study. All CT scans were performed with Toshiba Aquilion ONE TSX- 301C/7C 640 CT scanner according to the following parameters: slices thickness - 1mm, increment - 0.8mm, collimation - 80 x 0.5mm, exposure setting 120kV and 250mA, rotation time - 0.75 secs (center 800 HU, width 2000HU). The raw data of the identified CT scans were reconstructed to the bone algorithm, 1 mm slice thickness using OSIRIX, 64-bit DICOM viewer, analysed and recorded together with the relevant demographic information on a data collection sheet.

**Figure 1:**
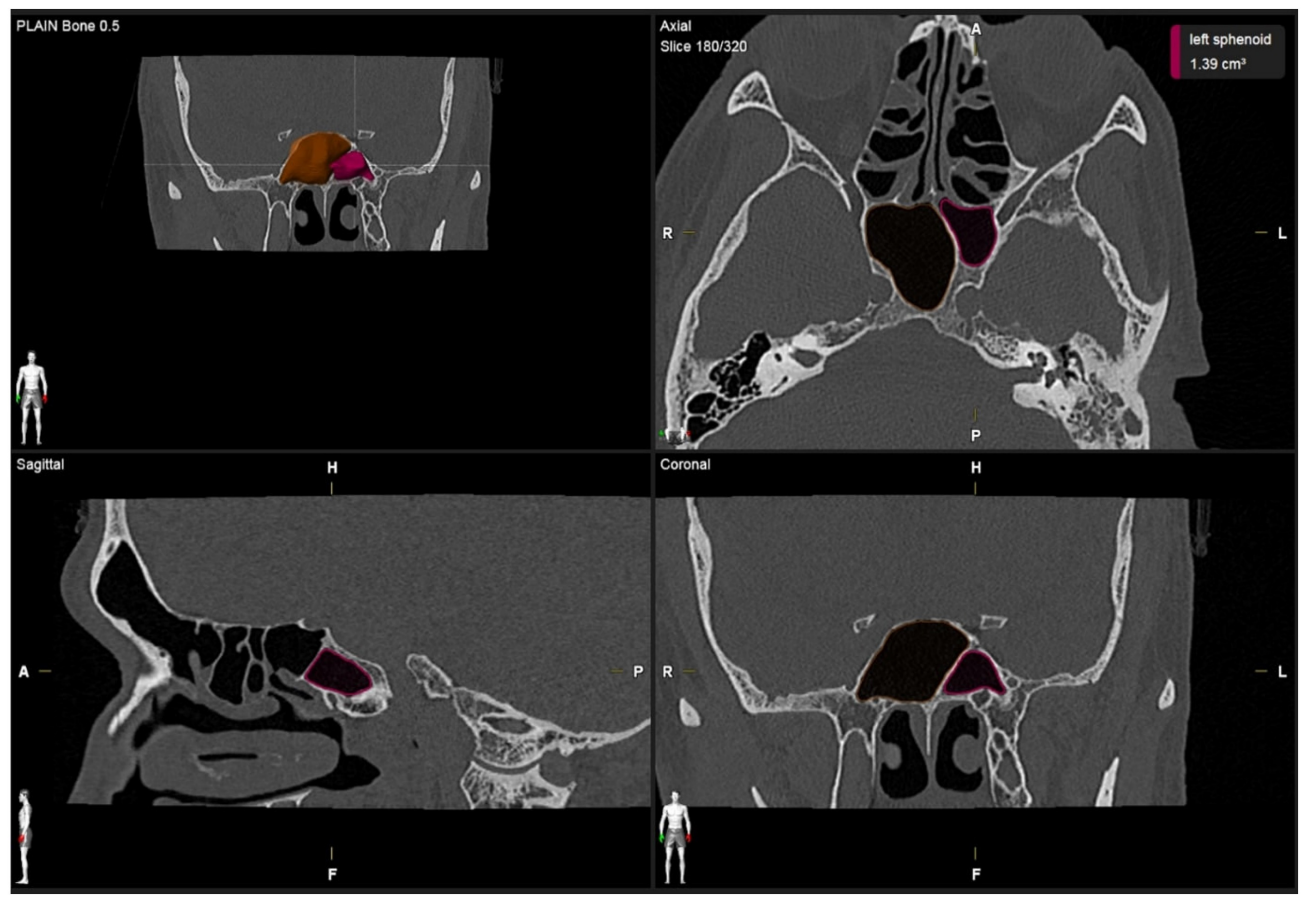
Volumetric measurement of the right and left sphenoid sinus.

**Figure 2:**
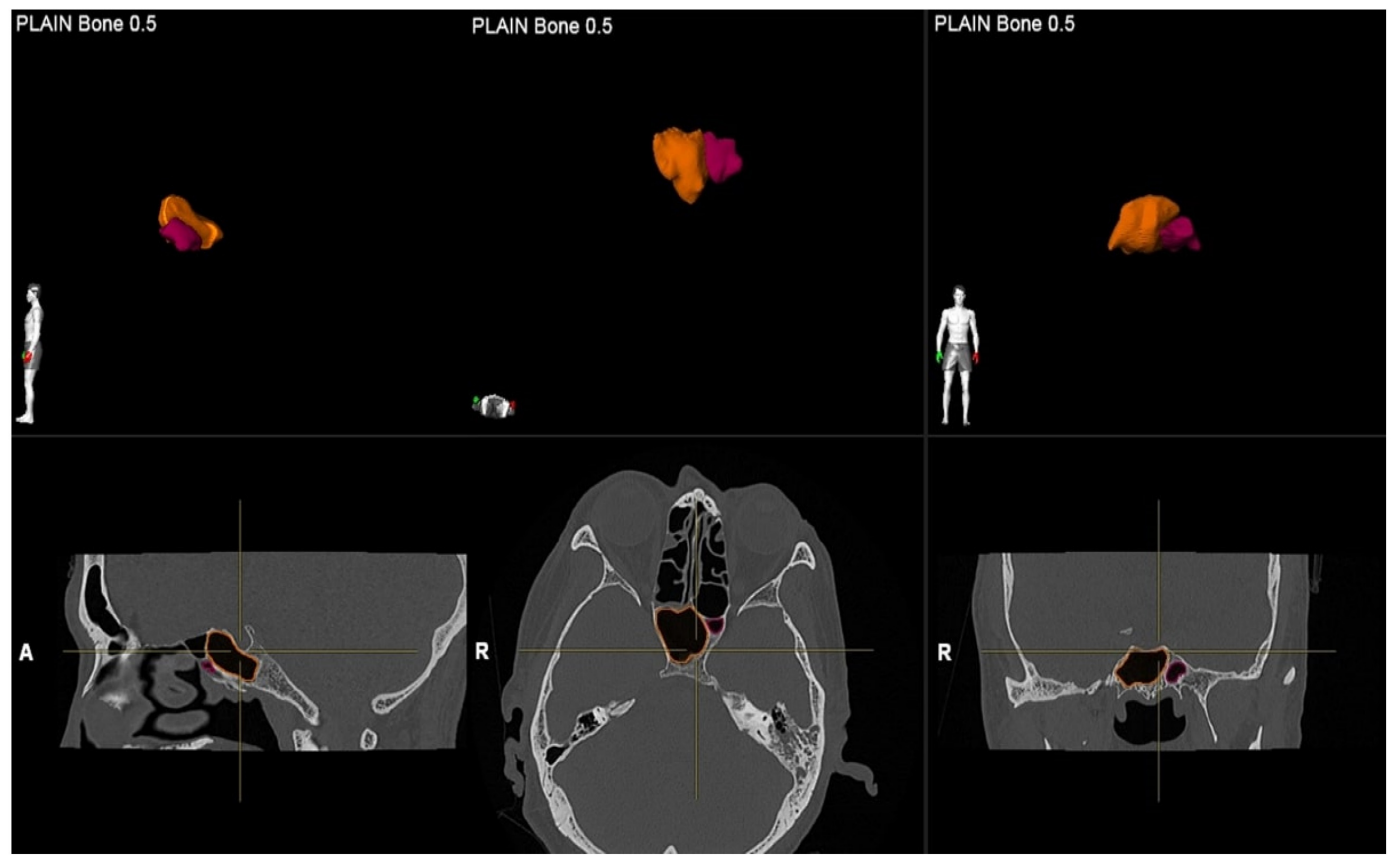
3D reconstruction in the sphenoid sinuses in all planes (sagittal, axial, and coronal).

A single investigator, an otolaryngologist with more than ten years of clinical experience, performed image segmentation by measuring the volume of the sphenoid sinus. For each patient, the volume of the sphenoid sinus was measured using commercial real-time segmentation software (Elements SmartBrush, Brainlab AG, Munich, Germany) on a personal computer. The software exploits parallel implementation of a sparse field level-set solver on the Graphics Processing Unit (GPU) by implementing the level-set algorithm and has been utilised in various clinical applications such as tumour localisation and estimation of tumour dimensions 15,16. The segmentation was achieved using a set of Hounsfield units in CT. The anatomical volumetric measurements were performed by means of this software in hand tracing. The lumen of the sinus was delineated. The lumen of the sinus was defined as the space within the bony walls of the sinus in all three planes (axial, coronal, and sagittal) (Figure 1). Within an automatically determined region of interest (ROI), segmentation was conducted by the 3D interpolation of the program itself. The software uses an inverse present method. The program reconstructs a 3D model of the sinus from the DICOM image sequence on which the volume was selected by cutting out the complementary areas of the air-filled area in the three dimensions manually; then, the volume was reconstructed and measured in cubic centimetres (cm^3^) by the software (Figure 2). Each side of the sphenoid sinus (right and left) were measured separately. The total sphenoid sinus volume was obtained based on the right and left sphenoid sinus volume summation. After 15 days, the measurements were repeated. All information was analysed using Statistical Package for Social Sciences (SPSS) software version 26.0.

### Statistical analysis

Statistical data analysis was performed with SPSS 26.0 for Windows (SPSS Inc., Chicago, IL). The Shapiro-Wilk Test was used to test the normality. The numeric variables were presented as total numbers (n) and mean ± standard deviation values, whereas number (n) and frequencies (%) were used to present categorical variables. Abnormally distributed data were represented by median and 5th - 95th percentiles range. A two-way mixed effect model based on a single rating assessed the intra-rater repeatability of the sphenoid sinus volume. Mean estimations and 95% confidence intervals (CI) were reported for each intraclass correlation coefficient (ICC). The association of the PNS development between gender and three race cohorts was performed using the Mann-Whitney U test and Kruskal Wallis test, respectively, and the corresponding *p* values were obtained. The *p* - value was considered significant when < 0.05. The correlation between sinus volume and age was assessed using Spearman's rank coefficient.

## Results

**Figure 3:**
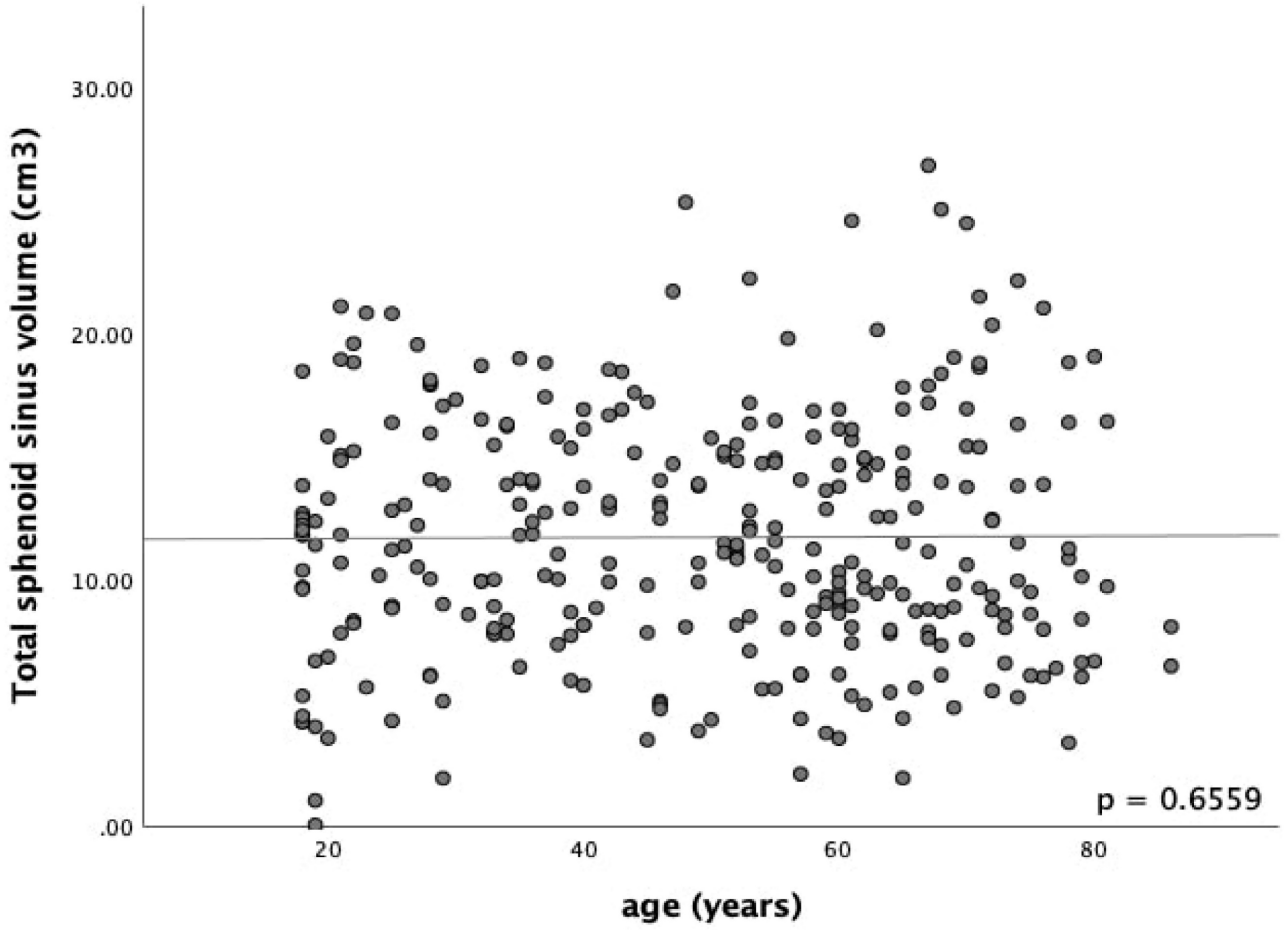
Scatter plot of the correlation between the age and the total volume of sphenoid sinus. Spearman's rho correlation coefficient was used to assess the relationship between the age and the total sphenoid sinus volume. No significant correlation was found (*r*_s_ = -.026, *p* = .6559, *N* = 304, *p* value = 0.6559).

A total of 304 patients, comprised of 167 males and 137 females, who met the study inclusion criteria were included in the analysis. The mean (± SD) age of the study population was 49.3 (± 18.6) years, and the age of the patients ranged from 18 to 86 years. The Malay ethnics constituted 190 out of 304 out of the sample size (62.5%), followed by Chinese, 87 (28.6%), and Indian, 27 (8.9%). The ICC for the intra-rater repeatability was close to 1, indicating an excellent agreement between the two measurements (Table 1). The age did not appear to be correlated with the volume changes of the sphenoid sinus, *r* s = -.026, *p* = .6559, *N* = 304 (Figure 3).

**Figure 4:**
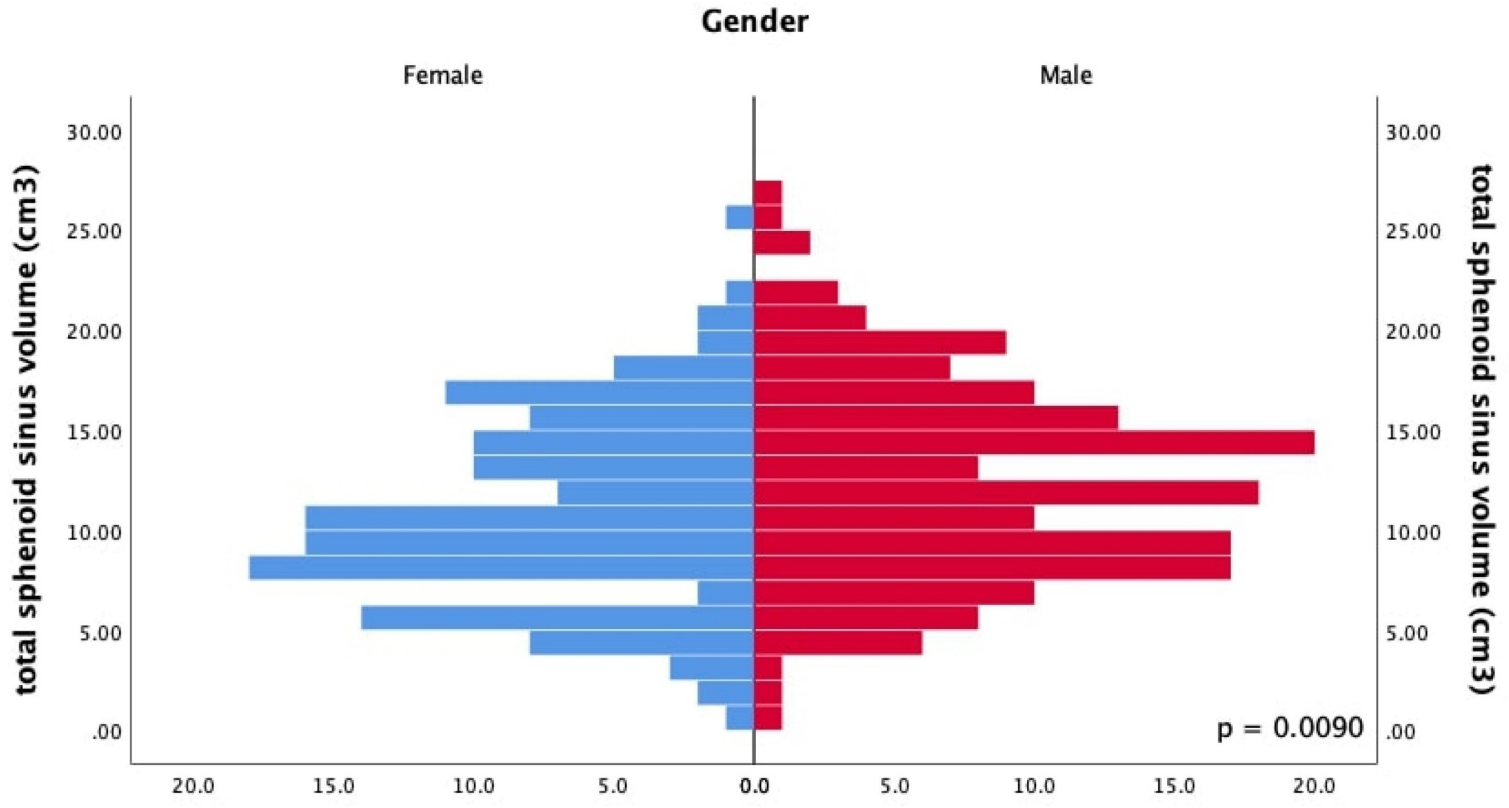
Histogram of total sphenoid sinus volume by gender. Male has a higher total sphenoid sinus volume than female. (*U* (*N*_male_ = 167, *N*_female_ = 137,) = 9449.00, *z* = -2.61, *p* = .0090).

Table 2 shows the sphenoid sinus volume among the males and females. Among the males, the volume of the right sphenoid sinus was 5.71 (0.66 - 11.95) cm^3^, and that of the left side was 6.12 (1.12 - 13.26) cm^3^. The total sphenoid sinus volume was 12.22 (4.93 - 21.09) cm^3^. Among the female, the volume of the right sphenoid sinus was 5.13 (0.74 - 11.42) cm^3^, and on the left, it was 5.36 (1.13 - 10.48) cm^3^. The total sphenoid sinus volume was 10.19 (3.75 - 18.72) cm^3^. In a comparison of males and females, the total volume of sphenoid sinus of the male gender had shown to be larger compared to the counterpart, *U* (*N* male = 167, *N* female = 137,) = 9449.00, *z* = -2.61, *p* = .0090 (Figure 4).

**Figure 5:**
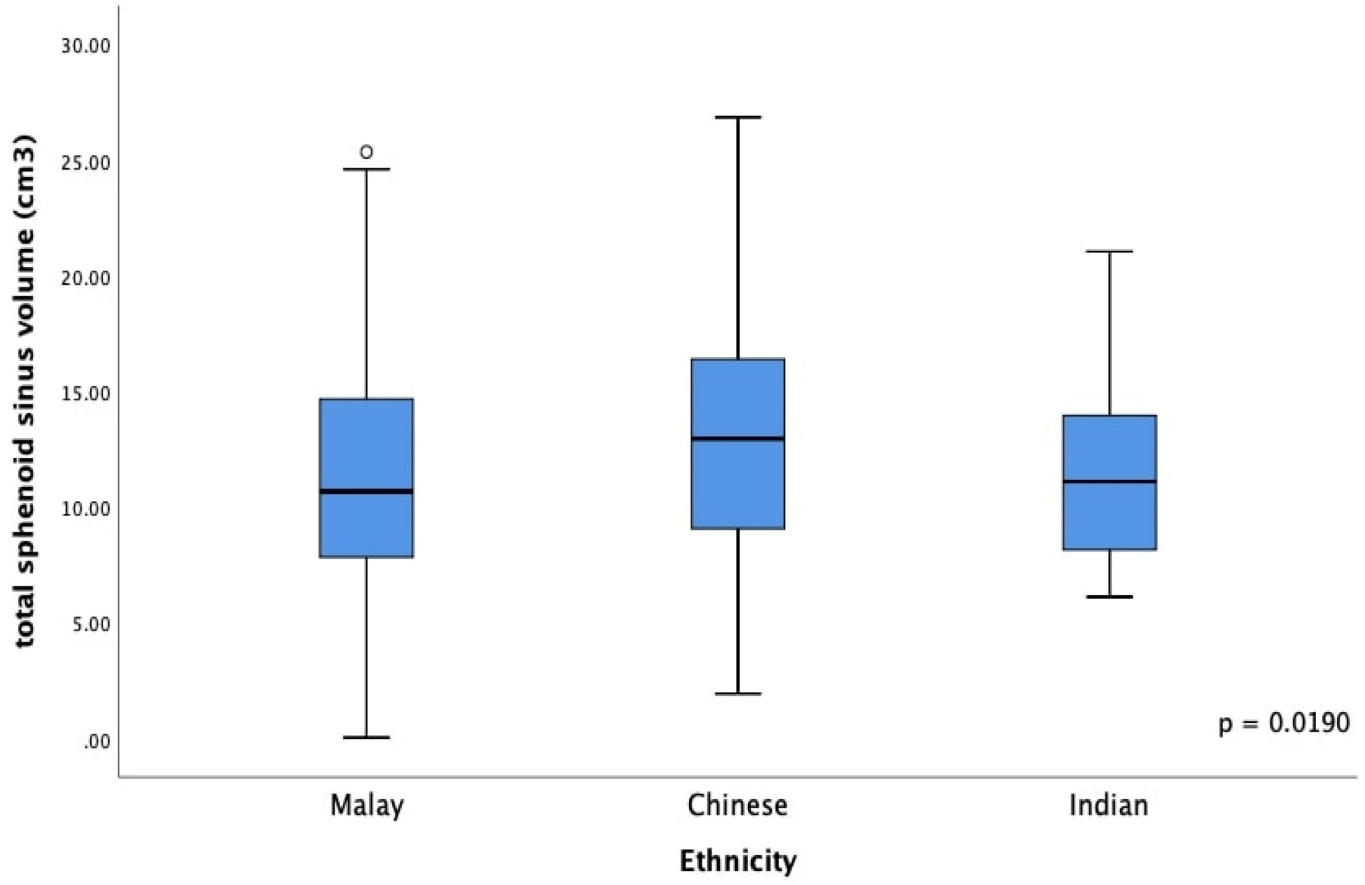
Boxplot of total sphenoid sinus volume among different races. Ethnicity has an influence in the total sphenoid sinus volume (*p* value = 0.0190).

Table 3 compares the total sphenoid sinus volume among different races. Among the Malays, the volume of the right sphenoid sinus was 5.41 (0.73 - 10.74) cm^3^, and that of the left side was 5.43 (0.98 - 12.37) cm^3^. The total sphenoid sinus volume was 10.68 (4.13 - 19.25) cm^3^. The right sphenoid sinus volume among Chinese was 5.82 (0.65 - 13.40) cm^3^, while the left side was 7.05 (1.22 - 12.81) cm^3^. The total sphenoid sinus volume was 12.96 (4.62 - 22.21) cm^3^. The right and left sphenoid sinus volume among Indians was 5.24 (1.35 - 13.17) cm^3^ and 4.86 (1.58 - 11.30) cm^3^, respectively. The total sphenoid sinus volume was 11.11 (6.13 - 20.26) cm^3^. The ethnicity was found to be significant in influencing the total sphenoid sinus volume, *H* (2) = 7.93, *p* = .0190 (Figure 5). Post hoc Mann Whitney U test revealed that the Chinese had a larger sphenoid sinus than the Malays, *U* (*N* Malay = 190, *N* Chinese = 87,) = 6553.00, *z* = -2.77, *p* = .0057. However, similar results were not found from other comparisons with p > .05. [Malay versus Indian = *U* (*N* Malay = 190, *N* Indian = 27,) = 2435.00, *z* = -.43, *p* = .6702; Chinese versus Indian* U* (*N* Chinese = 87, *N* Indian = 27,) = 951.00, *z* = -1.49, *p* = .1363].

## Discussion

The study of the PNS has become a valuable alternative method cohesively for dependable radiographic studies that promote body identification 2-4. The sphenoid sinus possesses tremendous morphological variability and degree of pneumatisation. However, less attention was given to the sphenoid sinus in the past due to the poor accessibility to its deep-seated anatomical location. Because of the superimposition of adjacent bony structures, conventional skull radiographies failed to provide an accurate visualisation when compared to the maxillary and frontal sinuses. Such constraint is eliminated with the utilisation of CT, which is today regarded as the gold standard imaging modality to study the PNS. Embryologically, the sphenoid bone is devoid of air at birth and comprises erythropoietic marrow instead. The sphenoid sinus is formed from the invagination or posterior extension of the nasal capsule. Pneumatization only begins in the first year of life and continues until the end of the third decade. It progresses in an inferior posterolateral direction with a variant degree 14,17.

In a study of 214 patients with ages ranging from 1 to 80 years, Yonetsu et al. studied the aeration of sphenoid sinuses of different age groups with CT. They found a reduction of the sinus volume in the late stages of life 18. Emirzeoglu et al. estimated the volume of paranasal sinuses of 77 adults serologically with CT. He observed a negative correlation between the total volume of all four sinuses and age. However, the correlation did not appear significant with individual sinuses 19. Cohen et al. found that older patients had a significantly lower volume of maxillary and sphenoid sinuses 20.

On the other hand, Andrianakis A, et al. examined the sphenoid sinus volume and its anatomical variants of pneumatisation by using a sphenoid sinus cast made of Quadro functional hydrophilic addition reaction silicon on 50 elderly human cadavers. They did not find age-related volume degeneration (*p* = .707) 20. Similarly, our study did not find any significant correlation between age and sinus volume in adults (cc = - .026, *p* = .6559).

Oliveria et al. observed that the left sphenoid sinus volume was higher than the right side 21. Our study found a similar finding of a higher volume sphenoid sinus on the left than the right side, and such observation was the same across gender. Studies in the past generally observed a larger sphenoid sinus volume in males than in females. The statistical correlation between sinus volume with gender, however, has been inconsistently reported 18-22. These discrepancies could be attributed to several influencing factors, such as the method of measurement, age, and demographic factors 18-24. Table 4 illustrates the correlation between gender and sinus volume from other studies 18-25. Our study revealed a significant difference in total sphenoid sinus volume between sex (*p* =.0090), indicating that the sphenoid sinus volume may function as an adjunctive measurement in gender determination.

It is of interest to find that studies in the western hemisphere demonstrated mean sphenoid sinus volume of not more than 12 cm^3^, with a reported maximum sphenoid sinus of not more than 25 cm^3^ 12,18-25. The present study's mean sphenoid sinus volume was 11.73 ± 4.92 cm^3^, with a maximum total sphenoid sinus volume of 26.85 cm^3^. Such a finding resonated with a study by Li et al. in China, of which the smallest mean sphenoid sinus volume among Asians was reported to be 11.16 ± 1.6 cm^3^ while the largest was 25.03 ± 2.21 cm^3^ 25. In comparing the sphenoid sinus volume between different ethnicities, the present study illustrated that race influences the sinus's size (*p* =.0190).

There are a few limitations in our study. We retrospectively analysed the CT images of living subjects, while post-mortem degradation of the skull can be consequent, particularly in traumatic death or incineration. Additionally, the study was conducted retrospectively in a single centre with limited samples and may not reflect the overall population. Nonetheless, the present study provides a scale model for volumetric measurement of the sphenoid sinus based on CT in the local community. To the authors' knowledge, the present study provides a scale model on CT volumetric measurement of the sphenoid sinus based on the local community. It represents the first volumetric study to correlate the SEA population's sphenoid sinus volume among different ethnicities. The results of this study provide a glimpse of using sphenoid sinus volume for body identification.

## Conclusion

The present study concluded that the sphenoid sinus volume in males was larger than in females, and this difference was statistically significant (*p* = .0090). It was also shown that race influences sinus volume (*p* = .0190). No correlation between the age and size of the sinus was identified. The authors ascertained the measurement of sphenoid sinuses volume as a possible adjunctive method in the forensic study. The sphenoid sinus is well protected within the sphenoid body and remains intact, although the skull and other bony structures may be disfigured or severely degraded. The result in gender variation of this study is comparable. Further studies with a larger sample size are needed to ensure the method is conclusive and achieves standardization.

## Figures and Tables

**Table 1:**
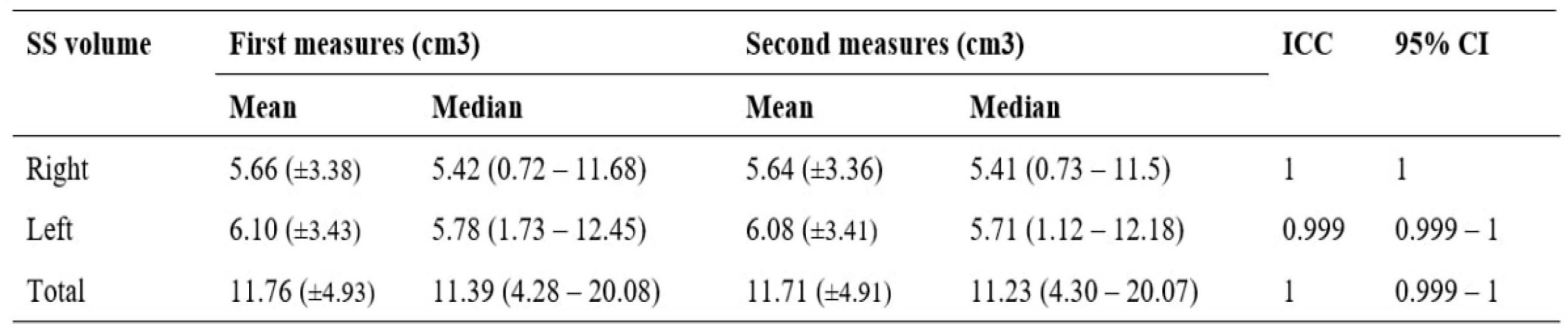
Means with standard deviation (SD) and median of sphenoid sinus (SS) volume

A two-way mixed effect model based on single rating assessed the intra-rater repeatability of the sphenoid sinus volume. Mean estimations along with 95% confidence intervals (CI) was reported for each intraclass correlation coefficients (ICC).

**Table 2:**
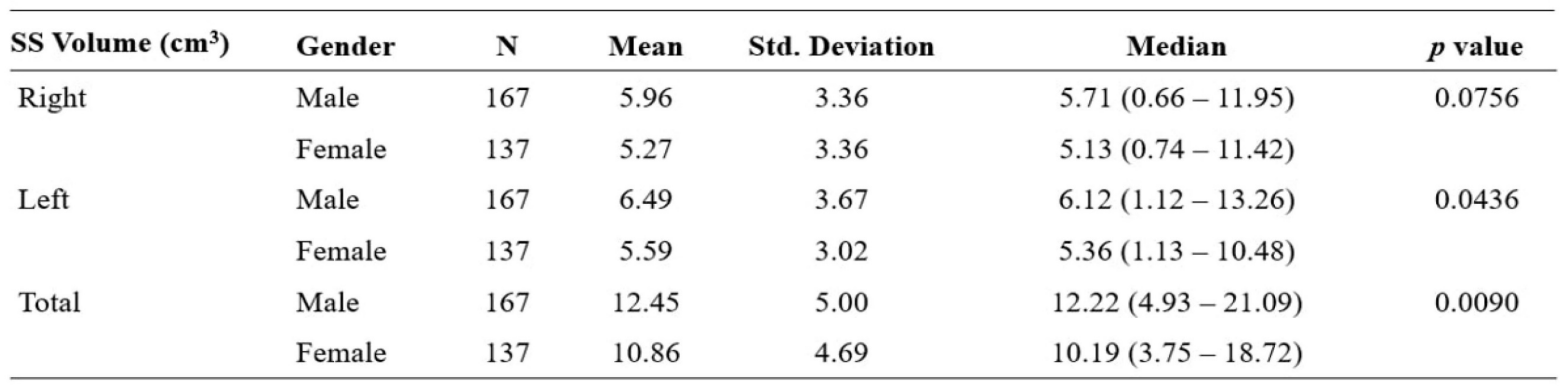
Sphenoid sinus (SS) volume among genders; Mann Whitney U test was performed to investigate the relationship between gender the sphenoid sinus volume

The total volume of sphenoid sinus of male gender had shown to be larger compared to the counterpart, *U* (*N*_male_ = 167, *N*_female_ = 137,) = 9449.00, *z* = -2.61, *p* = .0090; Std. Deviation = standard deviation.

**Table 3:**
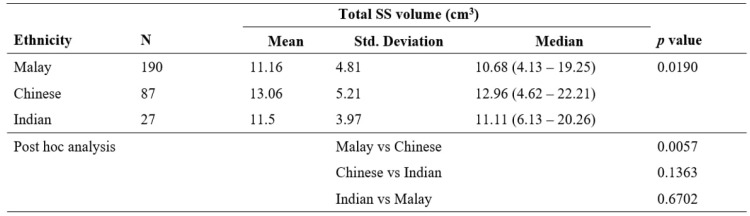
Mean with Standard deviation (Std. Deviation) and median of total sphenoid sinus (SS) volume among three races

Kruskal Wallis test showed significant association between ethnicity and total SS volume, *H* (2) = 7.93, *p* = .0190. Post hoc analysis was performed with Mann Whitney U test; Std. Deviation = standard deviation; vs = versus.

**Table 4:**
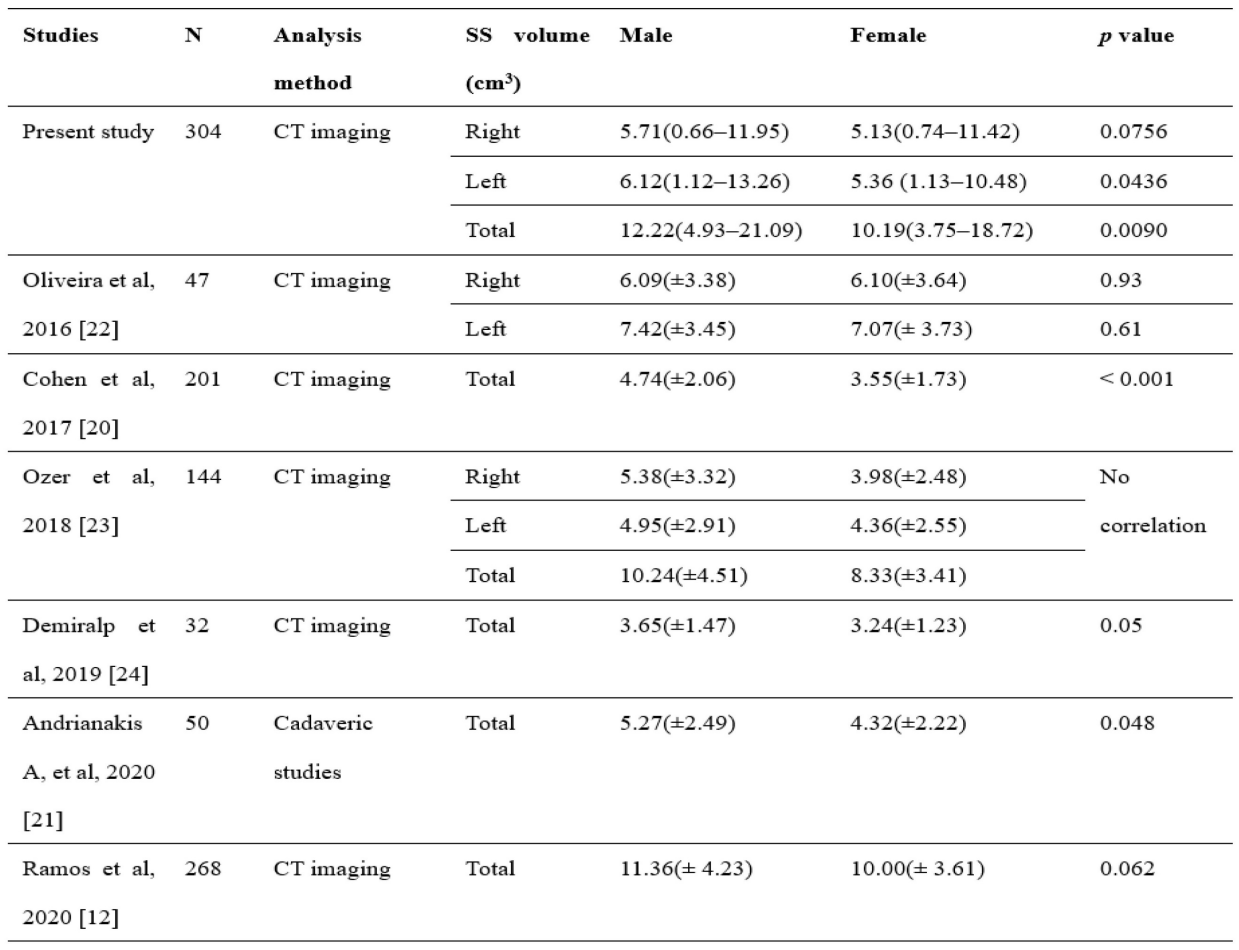
Comparison of study of evaluation of sphenoid sinus (SS) volume between gender
